# Functional Significance of Interprosthetic Joint Motion in Bipolar Hemiarthroplasty for Femoral Neck Fractures

**DOI:** 10.7759/cureus.107238

**Published:** 2026-04-17

**Authors:** Sitansu S Samantaray, Karthik Chavan, NK Sharath, Abhay Surjuse, Abhiram TV, Rishabh Jaiswal, Amol Godse, Pravin Londhe, Amit Supe, Aravind Chanal

**Affiliations:** 1 Department of Orthopaedics and Traumatology, Byramjee Jeejeebhoy Government Medical College and Sassoon General Hospital, Pune, IND; 2 Department of Orthopaedics and Traumatology, Grant Government Medical College and Sir JJ Group of Hospitals, Mumbai, IND; 3 Department of Orthopaedics and Traumatology, Indira Gandhi Government Medical College and Hospital, Nagpur, IND; 4 Department of Orthopaedics, Seth GS Medical College and KEM Hospital, Mumbai, IND

**Keywords:** bipolar hip hemiarthroplasty, fracture neck of femur, functional outcome, harris hip score, hip arthroplasty, interprosthetic joint movement, radiological assessment

## Abstract

Background: Fracture neck of femur is a common injury in the elderly and is frequently treated with bipolar hip hemiarthroplasty. The proposed advantage of a bipolar prosthesis is dual articulation, with motion occurring both at the prosthesis-acetabulum interface and at the interprosthetic joint (IPJ). However, previous studies suggest that the bipolar prosthesis may progressively behave as a unipolar device due to reduced IPJ mobility. The relationship between IPJ movement and functional outcomes remains controversial.

Objective: To radiologically assess IPJ movement in bipolar hip hemiarthroplasty performed for fracture neck of femur and to correlate these findings with functional outcomes at six months follow-up.

Methods: This observational retrospective study was conducted at a tertiary care center and included patients who underwent bipolar hip hemiarthroplasty for fracture neck of femur. Radiological assessment of IPJ movement was performed using standard anteroposterior radiographs obtained in the immediate postoperative period and at six months follow-up. A total of 60 patients with displaced femoral neck fractures were included. The proportion of motion occurring at the IPJ was measured relative to total hip movement. Functional outcomes were assessed using the Harris Hip Score (HHS), and correlations between radiological findings and functional outcomes were analyzed.

Results: Radiological evaluation demonstrated variable IPJ mobility among patients at six months of follow-up. A decline in IPJ movement was observed in a proportion of cases, indicating a tendency toward unipolar function. Functional assessment revealed predominantly good to excellent outcomes. Patients with preserved IPJ movement demonstrated better HHSs compared to those with reduced or absent IPJ mobility, showing a positive correlation between maintained IPJ motion and improved functional outcomes.

Conclusion: IPJ mobility in bipolar hip hemiarthroplasty shows variability at six months following surgery for fracture neck of femur. Preservation of the IPJ movement is associated with superior functional outcomes. Radiological assessment of IPJ movement serves as a useful tool in evaluating prosthesis behavior and predicting functional performance after bipolar hip hemiarthroplasty.

## Introduction

Femoral neck fractures represent one of the most consequential orthopedic problems affecting the aging population, contributing substantially to morbidity, mortality, and healthcare expenditure worldwide [1]. By 2050, the global prevalence of these injuries is expected to triple due to rising life expectancy and increased rates of osteoporosis [2,3]. Although the optimal management of displaced femoral neck fractures in older patients remains a topic of debate, prosthetic reconstruction has become the preferred strategy because it facilitates early weight-bearing and circumvents complications such as avascular necrosis and nonunion [4,5].

James E. Bateman and Gilberty first wrote about bipolar hip hemiarthroplasty in 1974. It was a big step forward from the old unipolar (Moore/Thompson) implants [5]. The bipolar construct incorporates two bearing surfaces: an inner articulation between the prosthetic head and a high-density or ultra-high molecular weight polyethylene (UHMWPE) liner, and an outer articulation between the metallic cup and the native cartilage. This dual-motion design aims to distribute movement across both interfaces, thereby limiting acetabular wear, reducing the risk of erosion and protrusion, and extending implant longevity [6,7].

Despite these theoretical merits, long-term investigations have shown that the interprosthetic joint (IPJ) progressively loses mobility, eventually causing the prosthesis to function as a unipolar device [8,9]. This process is believed to stem from progressive fibrosis and the accumulation of polyethylene debris within the inner bearing. Studies employing fluoroscopy and plain radiography have reported wide variation in IPJ preservation rates, with some observing near-total rigidity as early as three months postoperatively [8]. Current literature has not thoroughly established how preserved IPJ motion influences functional recovery.

This retrospective observational study was designed to radiographically measure the degree of IPJ excursion at the immediate postoperative and six-month time points using the Bochner plain radiograph technique and to examine the relationship between these measurements and patient function as gauged by the Harris Hip Score (HHS). Secondary objectives included comparing findings with Indian and international published data and identifying factors that predict IPJ mobility retention.

## Materials and methods

After receiving clearance from the Institutional Ethics Committee, this retrospective observational study was carried out between January 2023 and June 2024 at the Department of Orthopaedics and Traumatology, Grant Government Medical College and Sir JJ Group of Hospitals, Mumbai, Maharashtra, India. All participants or their legally appointed representatives provided written informed consent.

The study comprised 60 patients over 55 years old who had displaced femoral neck fractures and underwent bipolar hemiarthroplasty. Individuals with open fractures, pathological fractures, ipsilateral lower limb injuries, previous hip surgery, preexisting hip pathology, bilateral fractures, and inadequate follow-up were not included. The sample size was derived using the formula n=Z²×p(1-p)/d², where Z is the standard normal deviation corresponding to a 95% confidence level (1.96), p is the predicted prevalence, set to 0.5, and d is the absolute precision set at 0.05. This calculation led to the conclusion that 60 patients were the necessary sample size. All patients underwent bipolar hemiarthroplasty using the SMPL bipolar prosthesis (ISO 13485-certified). No financial or material support was received from the manufacturer.

Radiographic assessment

**Figure 1:**
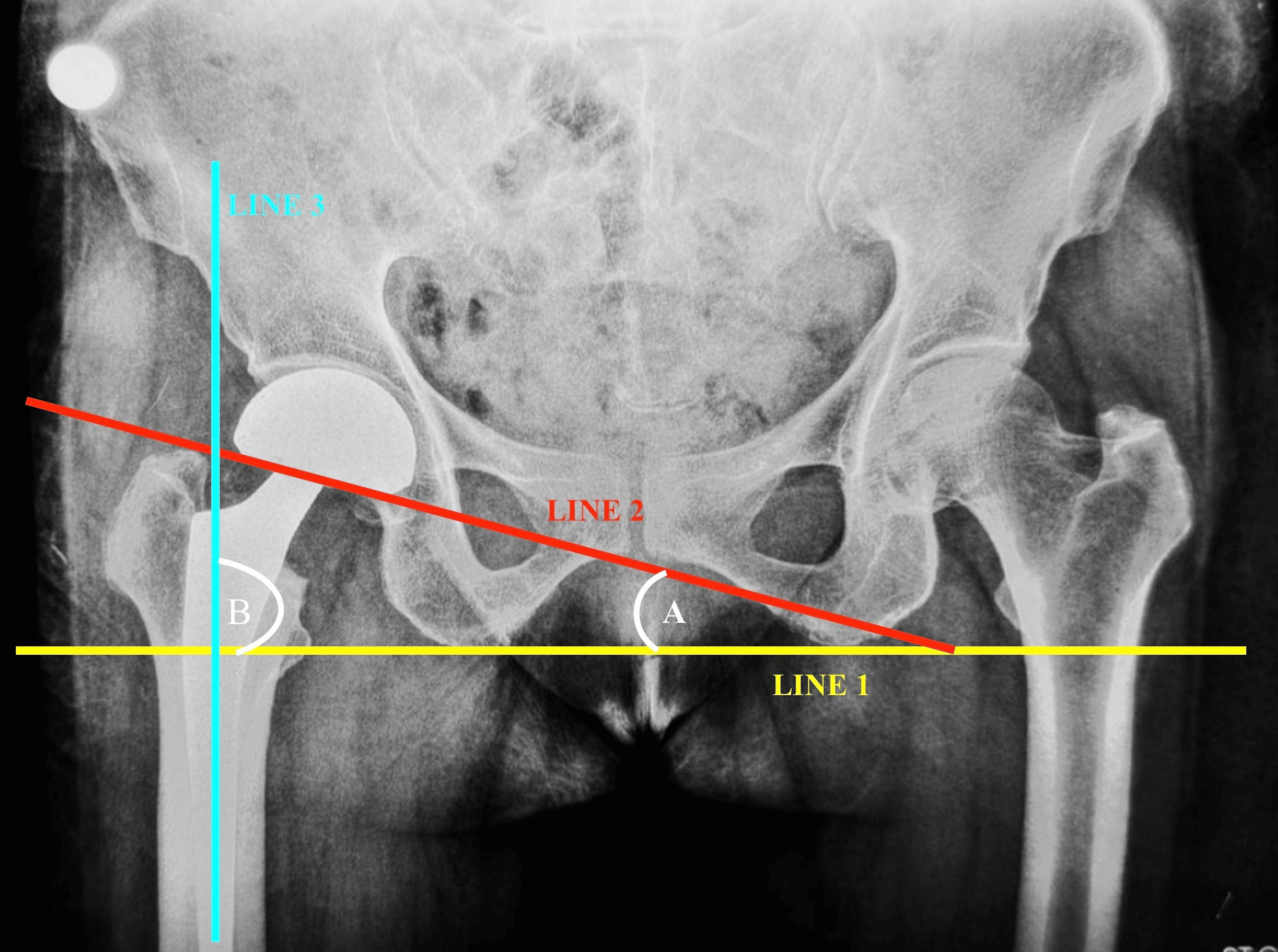
Neutral AP pelvic radiograph demonstrating reference lines and angular measurements (A and B) Line 1: a line drawn tangential to the most inferior aspects of the ischial tuberosities, which was used as a reference line. Line 2: Drawn along the inferior margin of the acetabular component, and Line 3: Drawn along the center of the long axis of the femoral stem. Angle A is defined as the point where the reference line intersects the line drawn from the inferior border of the acetabular component (line of ischial tuberosities). The ischial reference line and a line drawn across the middle of the long axis of the femoral stem intersect to generate angle B. AP, anteroposterior

**Figure 2:**
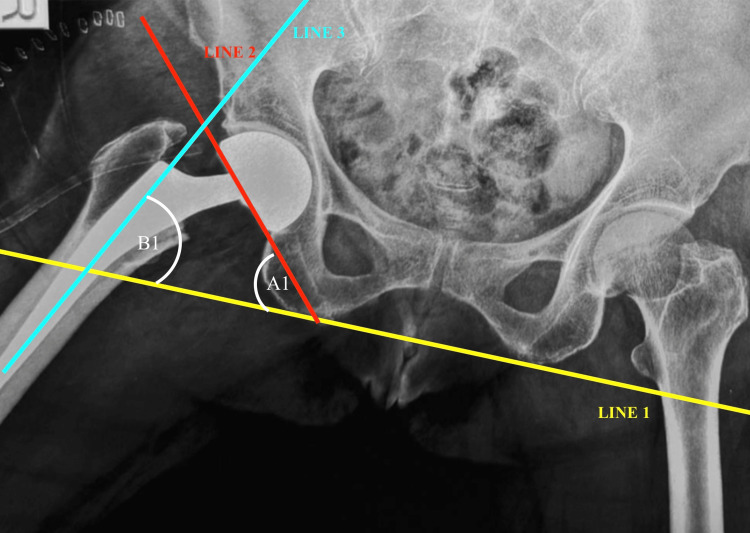
AP pelvic radiograph in maximum abduction showing corresponding angular measurements (A₁ and B₁) The same technique was performed on the maximal abduction anteroposterior radiograph, with the angles denoted as A1 and B1. AP, anteroposterior

Standardized anteroposterior (AP) pelvic radiographs obtained in neutral and maximal abduction positions were used to assess IPJ mobility (Figure 1 and Figure 2). All radiographs were retrieved from the Department of Radiology after obtaining the necessary institutional permissions. To maintain consistency, radiographs were taken with patients in the supine position, ensuring the pelvis was in a neutral orientation with both anterior superior iliac spines aligned to minimize rotation. The lower limbs were positioned with the patellae facing upward. For the abduction view, the operated limb was placed in maximum passive abduction tolerated by the patient. Radiographic measurements were performed using the technique described by Bochner et al. [10]. All measurements, including reference line construction and angular calculations, were carried out using Angulus software, which provides digital tools for precise and reproducible measurements. For analysis, three reference lines were defined: Line 1 was drawn along the inferior margin of the ischial tuberosities and served as the baseline reference; Line 2 was drawn along the inferior margin of the acetabular component; and Line 3 was drawn along the longitudinal axis of the femoral stem. The angle between Line 1 and Line 2 in the neutral and abduction positions was recorded as angle A and angle A₁, respectively, while the angle between Line 1 and Line 3 in the corresponding positions was recorded as angle B and angle B₁. These measurements were used to derive the key parameters of interest. Total hip abduction (B₂) was calculated as the difference between B₁ and B, and outer bearing motion (A₂) as the difference between A₁ and A. IPJ excursion was then calculated as B₂-A₂. The relative contributions of motion were expressed as percentages, with outer bearing contribution calculated as (A₂/B₂)×100 and IPJ contribution calculated as ((B₂-A₂)/B₂)×100. To reduce observer bias, all measurements were performed by a single blinded observer, and each measurement was repeated, with the mean value used for analysis. The use of digital measurement software further improved consistency and minimized observer-dependent variability.

Fracture classification

Based on the degree of displacement on anteroposterior radiographs, fractures were categorized using Garden's classification [11]. Type I fractures are incomplete or valgus-impacted; Type II fractures are complete without displacement; Type III fractures are complete with partial displacement and trabecular alignment disruption; and Type IV fractures are completely displaced with loss of continuity and realignment of the femoral head with the acetabulum.

Definition of IPJ motion groups

Patients were divided into two groups based on the percentage of IPJ motion: those with IPJ contribution <25% and those with IPJ contribution ≥25%. In line with other research, where ≥25% interprosthetic motion indicated retention of inner bearing mobility, a criterion of 25% was utilized to identify a functionally mobile bipolar articulation [12].

Functional assessment

The HHS, as first reported by Harris WH (1969) [13], was used to evaluate functional results at six months. Excellent (90-100), good (80-89), fair (70-79), and poor (<70) were the categories for scores.

Correlation and comparative analysis

The relationship between IPJ motion and functional outcome was evaluated using both continuous and categorical methods. Pearson’s correlation was used to assess the association between IPJ contribution (%) and HHS. Patients were also divided into two groups based on IPJ contribution (<25% and ≥25%), and their functional outcomes were compared. In addition, a 2×2 table was used to examine how HHS categories (≥90 vs. <90) were distributed across these groups, with the chi-square test used to assess this association.

Statistical analysis

MINITAB software was used for statistical analysis. The mean±SD was used to express continuous variables. Measurements over time were compared using a paired t-test, and group comparisons were done using an independent t-test. Chi-square and Pearson's correlation tests were used as necessary. Statistical significance was defined as a p-value of <0.05.

## Results

Demographic and clinical characteristics

The study included a total of 60 patients. The majority of patients were between the ages of 65 and 74 (36.67%), followed by those between the ages of 55 and 64 (33.33%) and 75 and 84 (26.66%). Only a small percentage of patients were older than 85 (3.34%). With 56.66% of the cohort being male and 43.34% being female, there was a small male majority.

According to published research [14,15], the majority of injuries (83.33%) were due to domestic falls, followed by road traffic accidents accounting for 16.67%. Transcervical fractures accounted for 50% of all fractures, with subcapital fractures second at 33.33% and basic fractures at 16.67%. According to Garden's classification, the most frequent fractures were Type IV (46.67%), followed by Type III (36.67%) and Type II (16.67%) (Table 1).

**Table 1:** Demographic and clinical characteristics of study participants (n=60)

Variables	Category	Frequency (n)	Percentage (%)
Age groups (in years)	55-64	20	33.33
	65-74	22	36.67
	75-84	16	26.66
	≥85	2	3.34
Sex	Male	34	56.66
	Female	26	43.34
Mode of injury	Domestic fall	50	83.33
	Road traffic accident	10	16.67
Fracture type (anatomical)	Basicervical	10	16.67
	Transcervical	30	50
	Subcapital	20	33.33
Garden’s classification	Type II	10	16.67
	Type III	22	36.67
	Type IV	28	46.67

Radiographic assessment of IPJ excursion

The total hip abduction (B2) increased significantly from 17.40±4.58° immediately after surgery to 25.30±2.77° at six months, according to radiographic analysis (mean difference: 7.90°, p<0.001). Similarly, there was a significant increase in outer bearing motion (A2) from 8.10±4.65° to 17.90±2.62° (mean difference: 9.80°, p<0.001). Over the same period, IPJ excursion significantly decreased from 9.30±5.58° to 7.40±1.81° (mean difference: -1.90°, p=0.02), suggesting a decrease in inner bearing motion.

A significant change in the motion distribution was found by analyzing the percentage contribution. While IPJ motion decreased from 53.45±18.72% to 29.25±8.96% (mean difference: -24.20%, p<0.001), outer bearing motion (A2%) increased from 46.55±18.72% to 70.75±8.96% (mean difference: 24.20%, p<0.001). A progressive shift toward outer-bearing-dominant motion was indicated by all observed changes being statistically significant, with 95% CI not crossing zero (Table 2).

**Table 2:** Radiographic assessment of IPJ excursion The B2 represents total hip abduction, and A2 represents outer bearing motion. The IPJ excursion was calculated as B2-A2. The percentage contributions were calculated as (A2/B2)×100 and ((B2-A2)/B2)×100. The values were expressed as mean±SD. Comparisons were performed using a paired t-test. The mean differences represent the change from immediately postoperative to six months. A 95% CI indicates the precision of estimates; p<0.05 was considered significant. IPJ, interprosthetic joint

Parameter	Immediate post-op (mean±SD)	6 months (mean±SD)	Mean difference	SD of differences SD(d)	Test statistic (t)	p-value	95% CI
B2 (°)	17.40±4.58	25.30±2.77	7.90	6.64	9.21	<0.001	(6.19, 9.61)
A2 (°)	8.10±4.65	17.90±2.62	9.80	7.00	10.84	<0.001	(7.99, 11.61)
IPJ excursion (B2-A2) (°)	9.30±5.58	7.40±1.81	-1.90	6.00	-2.45	≈0.02	(-3.45, -0.35)
A2 contribution (%)	46.55±18.72	70.75±8.96	24.20	26.34	7.12	<0.001	(17.40, 31.00)
IPJ contribution (%)	53.45±18.72	29.25±8.96	-24.20	26.34	-7.12	<0.001	(-31.00, -17.40)

Functional outcome based on IPJ excursion

The cohort's mean HHS at six months was 90.30±7.04. Patients with IPJ excursions ≥25% exhibited significantly superior functional outcomes compared to those with IPJ excursions <25%. The mean HHS was 92.86±4.14 in the IPJ ≥25% group and 84.33±8.93 in the IPJ <25% group, with a mean difference of 8.53 points (p=0.023). This suggests a large effect size (Cohen's d=1.43) and a statistically significant clinical association between increased IPJ motion and improved functional outcome (Table 3).

**Table 3:** HHS according to IPJ excursion at six months IPJ contribution (%) was calculated as ((B₂-A₂)/B₂)×100. Values are expressed as mean±SD. Between-group comparison was performed using an independent t-test. The IPJ <25% group was considered the reference. The 95% CI represents the precision of the mean difference. IPJ, interprosthetic joint; HHS, Harris Hip Score

IPJ motion group	N	Mean HHS±SD	Mean difference	Test statistic (t)	p-value	95% CI
IPJ <25%	18	84.33±8.93	Reference	-	-	-
IPJ ≥25%	42	92.86±4.14	8.53	3.14	0.023	(4.13, 12.93)

Association between IPJ contribution and functional outcome

Patients were divided into ≥90 and <90 groups according to their HHS and compared across IPJ contribution levels (<25% and ≥25%) to better understand the link between IPJ mobility and functional results. Higher functional scores were more common in patients with an IPJ contribution of at least 25%. This correlation was statistically significant (χ²=9.96, p=0.0001) (Table 4).

**Table 4:** Association between IPJ contribution and functional outcome Patients were categorized into ≥90 and <90 groups. The association was tested using the chi-square test (χ²=9.96, p=0.0001). HHS, Harris Hip Score; IPJ, interprosthetic joint

IPJ contribution	HHS ≥90	HHS <90	Total
≥25%	32	10	42
<25%	6	12	18
Total	38	22	60

Correlation between IPJ contribution and functional outcome

Higher IPJ mobility is linked to improved functional results, according to correlation analysis, which revealed a moderately favorable association between IPJ contribution and HHS (Pearson's r=0.548, p<0.001) (Table 5).

**Table 5:** Correlation between IPJ contribution and HHS The Pearson correlation coefficient was used to assess the relationship between IPJ contribution (%) and HHS. HHS, Harris Hip Score; IPJ, interprosthetic joint

Parameter	Value
Pearson’s r	0.548
Degrees of freedom (df)	58
p-value	<0.001

## Discussion

This study presents a contemporary radiographic and functional evaluation of bipolar hemiarthroplasty performed at an Indian tertiary care center, using a standardized plain radiograph methodology. The principal finding is a statistically significant decrease in IPJ excursion between the immediate postoperative period (53%) and the six-month review (29%), coinciding with a shift toward outer bearing predominance. Importantly, individuals who retained IPJ motion >25% at six months demonstrated significantly superior functional scores, as reflected in higher mean HHS values.

The observed decline in IPJ motion from 53% to 29% at six months aligns with findings in the existing literature. Verberne (1983) recorded only 16.9% residual IPJ motion at three months using a fluoroscopic evaluation of 20 patients fitted with the Variokopf prosthesis [8]. Bochner et al. (1988), employing the same radiographic technique used in the present study, found 19% IPJ motion at six months in 26 patients with the Bateman bipolar prosthesis [10]. Our findings indicate a relatively better-preserved IPJ at six months, possibly attributable to advancements in UHMWPE liner technology. The improved wear resistance of UHMWPE relative to older high-density polyethylene is likely to have delayed IPJ stiffening caused by debris-induced inflammation.

Phillips (1987), in a fluoroscopic investigation of 100 patients, reported that only 25% of femoral neck fracture patients maintained bipolar function at four years, compared to 80% of those treated for hip arthritis [9]. This disparity may reflect differences in acetabular surface quality: in fracture patients, the articular cartilage is intact and relatively smooth, generating low friction at the outer interface and thereby directing motion preferentially to the outer cup rather than the inner bearing. Conversely, in arthritic patients, a roughened acetabular surface increases outer interface resistance, preserving inner bearing motion for a longer period. Anil Kumar Rai et al. (2011) reported 33.74% IPJ motion at three months in an Indian cohort treated with the BHU bicentric bipolar implant, which declined to 25.66% at 18 months [16]. Our six-month IPJ value of 29% is similar but slightly lower. This difference is probably due to differences in the design of the prosthesis.

The functional outcomes observed in this study compare favorably with historical and contemporary benchmarks. Our results, which show 90% satisfactory outcomes (excellent plus good), are similar to those of Rathod A et al. (2024) [17] and better than those of Gerber F et al. (2025) [18] and Singh S et al. (2020) [19]. The absence of poor outcomes may reflect careful preoperative patient selection, consistency of surgical technique, and early rehabilitation protocols.

The statistically significant association between retained IPJ motion (>25%) and HHS (p=0.023) is a clinically meaningful finding. It indicates that a functionally effective bipolar prosthesis, one that preserves genuine dual-surface motion, translates into superior patient-reported outcomes. This reinforces the clinical rationale for optimizing bipolar prosthesis design to protect inner bearing function over time. The absence of significant effects from age (p=0.37) or sex (p=0.996) on HHS is consistent with prior published studies.

This study carries several limitations. The retrospective design and small sample of 60 patients limit statistical power and the generalizability of findings. A six-month follow-up provides only short-term data; IPJ behavior may evolve substantially at one, two, and five or more years. Plain radiography, even when standardized with the Bochner method, only shows a two-dimensional picture of how joints function in three dimensions. Fluoroscopic dynamic evaluation and gait laboratory analysis would provide richer kinematic information. The single-center setting further introduces the possibility of institution-specific bias.

## Conclusions

Bipolar hip hemiarthroplasty using the SMPL implant delivers excellent short-term functional results in elderly patients with femoral neck fractures, with 90% of participants attaining satisfactory outcomes at six months. The IPJ remains active at short-term follow-up, though a statistically significant progressive shift from inner-bearing to outer-bearing motion occurs between the immediate postoperative period and six months. Critically, patients who preserve IPJ excursions >25% at six months achieve significantly higher HHSs, highlighting the functional importance of maintaining inner bearing mobility. These findings support the adoption of modern UHMWPE-lined bipolar prostheses with optimally matched femoral head sizes to maximize IPJ preservation.
